# Microparticles from vascular endothelial growth factor pathway inhibitor-treated cancer patients mediate endothelial cell injury

**DOI:** 10.1093/cvr/cvz021

**Published:** 2019-02-11

**Authors:** Karla B Neves, Francisco J Rios, Robert Jones, Thomas Ronald Jeffry Evans, Augusto C Montezano, Rhian M Touyz

**Affiliations:** 1 Institute of Cardiovascular and Medical Sciences, University of Glasgow, 126 University Place, Glasgow, UK; 2 Beatson West of Scotland Cancer Centre, Glasgow, UK; 3 Cancer Research UK Glasgow Clinical Trials Unit, Glasgow, UK; 4 Institute of Cancer Sciences, University of Glasgow, Glasgow, UK

**Keywords:** VEGF, Hypertension, Microparticles, Cancer, Endothelial cell

## Abstract

Vascular endothelial growth factor pathway inhibitors (VEGFi), used as anti-angiogenic drugs to treat cancer are associated with cardiovascular toxicities through unknown molecular mechanisms. Endothelial cell-derived microparticles (ECMPs) are biomarkers of endothelial injury and are also functionally active since they influence downstream target cell signalling and function. We questioned whether microparticle (MP) status is altered in cancer patients treated with VEGFi and whether they influence endothelial cell function associated with vascular dysfunction. Plasma MPs were isolated from cancer patients before and after treatment with VEGFi (pazopanib, sunitinib, or sorafenib). Human aortic endothelial cells (HAECs) were stimulated with isolated MPs (10^6^ MPs/mL). Microparticle characterization was assessed by flow cytometry. Patients treated with VEGFi had significantly increased levels of plasma ECMP. Endothelial cells exposed to post-VEGFi treatment ECMPs induced an increase in pre-pro-ET-1 mRNA expression, corroborating the increase in endothelin-1 (ET-1) production in HAEC stimulated with vatalanib (VEGFi). Post-VEGFi treatment MPs increased generation of reactive oxygen species in HAEC, effects attenuated by ETA (BQ123) and ETB (BQ788) receptor blockers. VEGFi post-treatment MPs also increased phosphorylation of the inhibitory site of endothelial nitric oxide synthase (eNOS), decreased nitric oxide (NO), and increased ONOO^−^ levels in HAEC, responses inhibited by ETB receptor blockade. Additionally, gene expression of proinflammatory mediators was increased in HAEC exposed to post-treatment MPs, effects inhibited by BQ123 and BQ788. Our findings define novel molecular mechanism involving interplay between microparticles, the ET-1 system and endothelial cell pro-inflammatory and redox signalling, which may be important in cardiovascular toxicity and hypertension associated with VEGFi anti-cancer treatment. *New and noteworthy*: our novel data identify MPs as biomarkers of VEGFi-induced endothelial injury and important mediators of ET-1-sensitive redox-regulated pro-inflammatory signalling in effector endothelial cells, processes that may contribute to cardiovascular toxicity in VEGFi-treated cancer patients.

**This article is part of the Spotlight Issue on Cardio-oncology.**

## 1. Introduction

Vascular endothelial growth factor (VEGF) is an important growth factor produced by several cell types in the vascular and haematopoietic systems with crucial functions in vascular development and function. VEGF is also produced under pathological conditions by cancer cells and promotes formation of new blood vessels (angiogenesis), important in tumourigenesis. VEGFA is the most biologically active isoform^1^ and interacts with three tyrosine kinase receptors (VEGFR1-3). In endothelial cells, VEGFA through VEGFR1/2 initiates signalling through tyrosine kinases resulting in cell growth, migration, increased permeability and vasodilation, mediated via nitric oxide (NO), and prostacyclin.^1–4^ These are major processes involved in normal vasculogenesis and function, however, in a cancer environment increased VEGF signalling promotes uncontrolled angiogenesis, leading to tumour growth and metastasis.

VEGF inhibitors (VEGFi) are potent anti-angiogenic drugs that have had major impact as anti-cancer drugs in the treatment of solid tumours. They have prolonged survival in some cancer patients and are increasingly being used, either as monotherapy or as part of combination regimens, as first- and/or second-line treatment for several cancers, including renal cell, colorectal, and hepatocellular cancer.^5^ However, VEGFi anti-cancer therapy is associated with significant cardiovascular toxicity with almost all patients developing hypertension.^6–9^ VEGF inhibition is also associated with thromboembolic disease, left ventricular systolic dysfunction, heart failure, and myocardial ischaemia. Common to these cardiovascular pathologies is endothelial dysfunction and vascular injury. Potential mechanisms implicated in VEGFi-induced hypertension include increased endothelin-1 (ET-1) levels, renin–angiotensin system activation, endothelial cell apoptosis, and microvascular rarefaction.^6^^,^^8^^,^^9^ Recently, we demonstrated in *in vivo* and *in vitro* studies that vatalanib, a VEGFi, increased the generation of reactive oxygen species (ROS) in vascular cells and decreased activation of endothelial nitric oxide synthase (eNOS) and production of nitric oxide (NO) resulting in endothelial dysfunction and vascular hypercontractility in VEGFi-treated mice.^10^ Many cellular processes underlie these vascular changes including production of endothelial microparticles, which may have relevance in the context of angiogenesis, because circulating microparticles are associated with VEGF expression, microvascular density, and angiogenesis in oral cancer.^11^

Cell-derived microparticles are small membranous structures (0.1–1 μm) shed by eukaryotic cells upon cell activation or stress.^12^^,^^13^ They carry membrane markers and cytosolic molecules derived from parent cells including microRNAs, DNA, RNA, phospholipids, and proteins and are detected in the circulation in physiologic and pathologic conditions. Microparticles reflect the parental cell profile and accordingly are considered as biomarkers of activation status of the parent cell from which they derived. In cardiovascular diseases associated with vascular injury (hypertension, atherosclerosis, and coronary artery disease) circulating levels of endothelial cell-derived microparticles (ECMPs) are increased and appear to reflect endothelial cell activation and vascular dysfunction.^12^^,^^14^^,^^15^ In addition to their biomarker role, microparticles are biovectors that carry bioactive molecules, which have functional effects on effector target cells. Recent studies reported that microparticles directly affect endothelial function by increasing endothelial cell oxidative stress and inflammation, reducing NO production, promoting endothelial cell senescence, and stimulating platelet and monocyte endothelial adhesion.^16–19^

Considering the multiple characteristics of microparticles they may be considered as both prognostic biomarkers and pathogenic effectors in pathological conditions. In the present study, we questioned whether microparticle status is altered in cancer patients treated with VEGFi and whether microparticles from VEGFi-treated patients influence effector endothelial cells.

## 2. Methods

All experimental studies using human plasma samples comply with the Declaration of Helsinki and has full West of Scotland Research Ethics Committee approval (REC 10/S0704/18). Informed consent was obtained from all subjects.

### 2.1 Study population

The eligibility criteria for this study included: no prior tyrosine kinase inhibitor (TKI) treatment; no diagnosis of malignant disease; patients attending the Beatson West of Scotland Cancer Centre for treatment; over 18 years of age; no medical or psychiatric illness that would contraindicate blood donation. All patients gave signed informed consent prior to sample collection and study protocol aligned with the principles set out in the Declaration of Helsinki. The median age of patients was 64 years (39–86 years).

Forty-two patients were initially recruited into the study, however, due to various factors (failure to collect post-treatment samples, inadequate blood collection, patient died), samples from only 39 patients were fully studied where we were able to isolate microparticles before and after VEGFi treatment.

### 2.2 Blood samples

Blood samples were collected in heparinized tubes from cancer patients pre-treatment and post-treatment with VEGFi (pazopanib, sunitinib, or sorafenib) in heparinized tubes. Blood was centrifuged for 10 min at 2000 rpm at room temperature to obtain platelet-poor plasma supernatant. Plasma was collected and centrifuged for 20 min at 1500 *g* to obtain platelet-free plasma (PFP) supernatant. PFP was aliquoted and stored at −80°C. Clinical information is detailed in Supplementary material online, *Table S1*.

All studies comparing effects of pre-treatment and post-treatment microparticles (MPs) were always performed using the same set of patients since the main aim of this study was to compare the effects of isolated MPs before and after VEGFi-treatment.

### 2.3 Characterization of circulating microparticles

**Figure 1:**
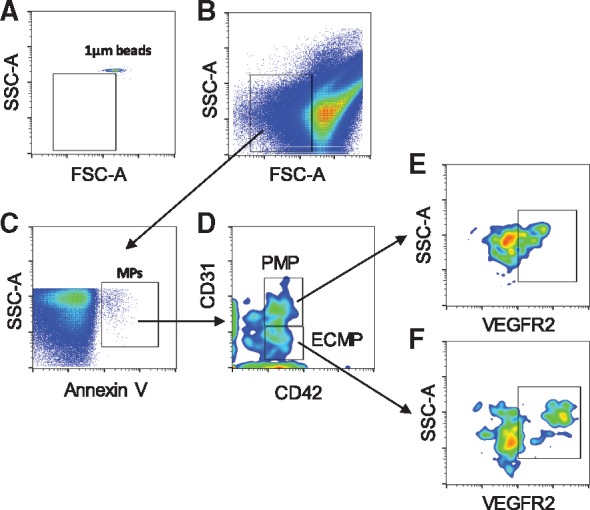
Representative flow cytometric multicolour gating strategy used to analyse number of MPs. (*A*) Location of 1 µm beads used to gate MP events according to size. (*B*) Representative of MPs events identified according to size (<1 µm). (*C*) Annexin V positive events (MPs). (*D*) PMPs (Annexin V+ CD31+ CD42b+) and ECMPs (Annexin V+ CD31+ CD42b-) populations were identified. (*E*, *F*) VEGFR2-expressing PMPs (Annexin V+ CD31+ CD42b+ CD309+) and ECMPs (Annexin V+ CD31+ CD42b- CD309+) were selected.

For microparticles studies, technical triplicates were performed for all samples. The PFP was thawed and 50 µL of PFP was incubated on ice in the dark for 30 min with 5 µL of anti-CD42b-FITC (final dilution 1:600) (clone HIP1, BD-Pharmingen) and 6 µL of anti-CD31-APC/Cy7 (final dilution 1:600) (clone WM59, Biolegend). Antibodies were carefully titrated in order to identify the best working concentration. Subsequently, 2.5 µL of Annexin-V-alexa 647 (Biolegend); 3 µL of 0.5 mol/L CaCl_2_, and 233.5 µL of NaCl 0.9% were added to make a final reaction volume of 300 µL. The specificity of antibodies was also checked using appropriate isotype controls—FITC Mouse IgG1 (clone MOPC-21, BD-Pharmingen), APC/Cy7 Mouse IgG1 (clone WM59, Biolegend). Every sample was processed separately by flow cytometry. We did not pool any samples. Microparticles were defined as particles identified between 0.1 µm and 1 µm in diameter as assessed by flow cytometry (FACS Canto II—BD Biosciences). Microparticle events were measured by FACS at constant medium flow for 4 min. Events were gated according to size (0.1–1 µm) at forward scatter and side scatter, both in log scale, using 1 µm size beads as a reference (#F13839; Molecular Probes, Life Technologies). Microparticles were characterized based on membrane marker expression: total microparticles were Annexin V+ (MPs), platelet microparticles (PMP) were Annexin V+ CD31+ CD42b+, and ECMPs were Annexin V+ CD31+ CD42b-. We also probed PMP and ECMP for VEGFR2 expression (PE mouse anti-human CD309, Clone 89106, BD-Pharmingen) (*Figure 1*). Experiments were also performed using the isotype control PE Mouse IgG1 (clone MOPC-21, BD-Pharmingen). MPs concentration was calculated using counting beads as a known standard with 1 mL of PFP used for normalization. FACS data were analysed using Flow Jo™ software (v10.0.7, Tree Star, Inc., OR, USA). The absolute numbers of microparticles, ECMPs and PMPs were calculated using the following formula: MP/mL = *A* × (*B*/*C*) × 20, where *A* = total number of MP events observed in the constant flow of 4 min; *B* = total number of counting beads added in the FACS tube before acquisition; *C* = total number of beads counted in the constant flow of 4 min; and 20 is the correction factor for 1 mL of plasma. These protocols were based on previous studies.^16^^,^^20–22^ Statistical analysis was performed on the mean value derived from the technical triplicates.

### 2.4 Isolation of microparticles

Plasma samples (1 mL) were placed in ultracentrifugation tubes and 2 mL of sterile/filtered PBS was added. Samples were centrifuged for 60 min at 20 000 *g* at 4°C (Optima™ TL Ultracentrifuge Beckman Coulter). The microparticle pellet was resuspended in lysis buffer (∼100 µL) for immunoblotting assays or in filtered and sterile PBS for cell stimulation (∼200 µL). MPs size was verified using the Nanosight technology (Nanosight^®^ LM14) in plasma samples diluted 1:1000 in sterile phosphatase buffer saline (PBS). Data were acquired using the Nanosight NTA 3.2 software.

### 2.5 Cell culture

For cell-based studies to assess effects of patients microparticles on endothelial cell signalling and function we used human aortic endothelial cell (HAEC) (ATCC^®^, Middlesex, UK; PCS-100-011). HAEC were cultured in Endothelial Cell Growth Medium (Promocell^®^) supplemented with Penicillin/Streptomycin (50 µg/mL) and Endothelial Cell Growth Medium Supplement (10 mL; Promocell^®^). For functional studies, confluent cells were made quiescent for 2 h in low-serum medium containing 0.5% foetal bovine serum (FBS) and stimulated with total microparticles (10^6^ MPs/mL) isolated from patients pre-VEGFi and post-VEGFi. Quiescent cells were used because this provides a system where molecular events can be studied in controlled conditions, where cells are not actively dividing. In addition, serum-free conditions avoid the potential for cell activation because serum contains growth factors and other proteins that trigger signalling and cell growth. This is especially relevant in our study, where we are interfering with growth factor (VEGF) signalling through VEGFi.

All studies comparing effects of pre-treatment and post-treatment MPs were performed using the same set of patients. In some experiments, cells were pre-treated with BQ123 (ETA receptor antagonist; 1 µmol/L) or BQ788 (ET_B_ receptor antagonist; 1 µmol/L).

### 2.6 Experimental protocols

#### 2.6.1 Nitric oxide production

Production of NO was determined using the NO fluorescent probe diacetate 4-amino-5-methylamino-2′,7′-difluorofluorescein diacetate (DAF-FM; Life Technologies, Molecular Probes, Paisley, UK). HAEC were load with DAF-FM diacetate (final concentration 5 µmol/L, 30 min) in serum free media, kept in the dark, and maintained at 37°C, as we previously described.^23^ In brief, cells were washed to remove exceeding probe. Medium was replaced and incubated for an additional 10 min to allow complete de-esterification of the intracellular diacetates. Cells were stimulated with total microparticles (10^6^ MPs/mL; 15 min) isolated from patients post-treatment with VEGFi in the absence or presence of BQ788 (ET_B_ antagonist, 1 µmol/L, 30 min preincubation). Cells were washed with phosphate-buffered saline (PBS) and harvested with mild trypsinization at 0.025%. Trypsin was inactivated with soybean trypsin inhibitor (0.025%) in PBS (1:1). After washing, the pellet was transferred to a black 96 well microplate (BD Falcon, Loughborough, UK). The DAF-FM fluorescence was assessed with a spectrofluorometer at excitation/emission wavelengths of 495/515 nm. Fluorescence intensity was normalized to the protein concentration and expressed as fluorescence emission/µg of protein.

#### 2.6.2 Lucigenin-enhanced chemiluminescence

Lucigenin-enhanced chemiluminescence assay was used to determine NADPH oxidase activity in HAEC as we previously described.^24^ Cells were stimulated with total microparticles (10^6^ MPs/mL) isolated from patients pre-VEGFi and post-VEGFi treatment for different time periods. In some experiments, 30 min prior to stimulation with MPs, cells were pre-incubated with BQ123 and BQ788. In brief, stimulated cells were washed with ice-cold PBS and harvested in lysis buffer (20 mmol/L of KH_2_PO_4_, 1 mmol/L of EGTA, 1 μg/mL of aprotinin, 1 μg/mL of leupeptin, 1 μg/mL of pepstatin, and 1 mmol/L of PMSF). The sample of 50 µL were added to a suspension containing 175 μl of assay buffer (50 mmol/L of KH_2_PO_4_, 1 mmol/L of EGTA, and 150 mmol/L of sucrose) and lucigenin (5 μmol/L). Luminescence was measured with a luminometer (AutoLumat LB 953, Berthold) before and after stimulation with NADPH (100 µmol/L). A buffer blank was subtracted from each reading. The results are expressed as a fold change in arbitrary units per milligram of protein, as measured by the BCA assay.

#### 2.6.3 Measurement of nitrotyrosine levels

Nitrotyrosine, a measure of peroxynitrite (ONOO^−^) formation, was assessed in cells using an ELISA kit (#ab113848, Abcam, Cambridge, UK), according to manufacturer’s instructions. The plate was read after stop the reaction by adding stop solution at absorbance of 450 nm using a microplate reader. Results were normalized to protein concentration.

#### 2.6.4 Quantitative real-time PCR

mRNA expression for pre-pro-ET-1, tumour necrosis factor-α (TNF-α), interleukin-6 (IL-6), monocytes chemoattractant protein-1 (MCP-1), inducible nitric oxide synthase (iNOS), cyclooxygenase-2 (COX-2), and vascular cell adhesion molecule-1 (VCAM-1) was measured by quantitative real-time PCR (Applied Biosystems) in non-stimulated (NS) or total microparticles-stimulated HAEC (8 h). In brief, total RNA was extracted from cells using QIAzol (Qiagen, Manchester, UK), treated with RNase-free DNAse I, and 2 μg of RNA was reverse transcribed in a reaction containing 100 μg/mL oligo-dT, 10 mmol/L of 2′-deoxynucleoside 5′-triphosphate, 5× First-Strand buffer, and 2 μL of 200-U reverse transcriptase. For real-time PCR amplification, 3 μL of each reverse transcription product were diluted in a reaction buffer containing 5 μL of SYBR Green PCR master mix and 300 nmol/L of primers in a final volume of 10 μL per sample. The reaction conditions consisted of two steps at 50°C for 2 min and 95°C for 2 min, followed by 40 cycles of three steps, 15-s denaturation at 95°C, 60-s annealing at 60°C, and 15 s at 72°C. The relative mRNA expression (target gene**/**Gapdh housekeeping gene) was calculated by the 2^−^^ΔΔCt^ method.^25^ Primers used are detailed in Supplementary material online, *Table S2*.

#### 2.6.5 Immunoblotting

Immunoblotting was used to assess ET-1 expression in isolated microparticles (1:1000, Santa Cruz #sc-517436, Middlesex, UK) and phosphorylation of eNOS (1:1000, Cell Signaling #9574, Hitchin, UK) in HAEC exposed to isolated MPs. In brief, cells were homogenized in lysis buffer [(in mmol/L) sodium pyrophosphate 50, NaF 50, NaCl 5, EDTA 5, EGTA 5, HEPES 10, Na_3_VO_4_ 2, PMSF 50, Triton 100 0.5%, and leupeptin/aprotinin/pepstatin 1 mg/mL] and sonicated for 5 s. Proteins (30 µg) were separated by electrophoresis on 12% and 7.5% SDS polyacrylamide gel, according to the molecular weight of the protein target, and transferred to a nitrocellulose membrane. Non-specific binding sites were blocked with 3% non-fatty dried milk in Tris-buffered saline solution with Tween (TBS-T) for 1 h at room temperature. Membranes were probed with anti-phosphorylated (Thr^495^) eNOS overnight at 4°C. Membranes were then washed in TBS-T and incubated with secondary fluorescence-coupled antibodies (LI-COR Biosciences, Cambridge, UK) in 1% BSA for 1 h. Membranes were scanned by an infrared laser scanner (Odyssey Clx, LI-COR Biosciences) and image analysis was performed using Image Studio™ Software. Protein expression levels were normalized to loading controls and expressed as absolute values or percentage (%) of the control. The signal from the bands of interest was plotted using GraphPad Prism^®^ software.

### 2.7 Effects of VEGFi on endothelial cell-derived ET-1 production

ET-1 levels were measured in media from HAEC exposed to vatalanib (100 nmol/L; 8 h) by colorimetric (532–535 nm) assay according to the manufacturer’s instructions (R&D Systems #QET00B, Abingdon, UK).

### 2.8 Statistical analysis

Statistical analysis was performed using GraphPad Prism 3.0 (GraphPad Software Inc., San Diego, CA, USA). Data are presented as means ± standard error of the mean (SEM). Groups were compared using Student’s *t*-test when differences between two groups were analysed. One-way analysis of variance (ANOVA) and the Tukey’s post-test were used to evaluate the statistical significance of the differences between three or more groups. Results of statistical tests with *P *<* *0.05 were considered significant.

## 3. Results

### 3.1 VEGF inhibition increases ECMP release

**Figure 2:**
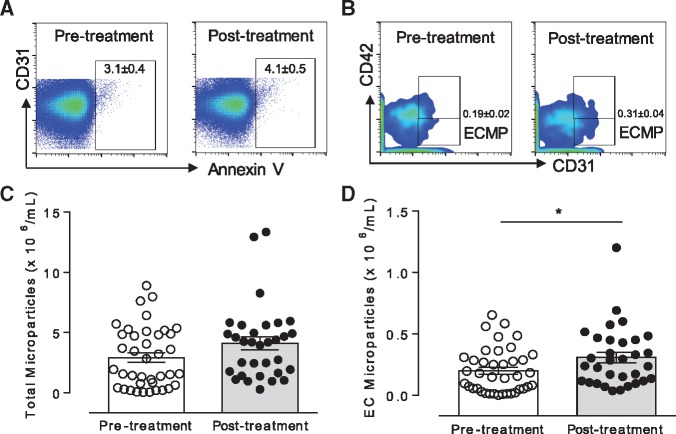
VEGFi increase ECMPs. (*A*) Representative of MPs events identified in plasma from patients pre-treatment and post-treatment with VEGFi. (*B*) Representative of ECMPs events identified pre-treatment and post-treatment with VEGFi. (*C*, *D*) Scatter plot graphs representing the total number of MPs and ECMPs, respectively identified in plasma pre-treatment and post-treatment with VEGFi (*n* = 32–39). Results represent the mean ± SEM. Data were analysed using *t*-test. *P < 0.05 vs. pre-treatment.

The strategy to define microparticles is shown in *Figure 1*. We show a representative from our PFP samples to exemplify our gating strategy to analyse our data. In *Figure 2*, we show a representative from a single donor for the pre-treatment and post-treatment bars. Each sample had a different flow cytometry run; no pooled samples were used for the flow cytometry experiments in this study.

Microparticle membranes possess phosphatidylserine that specifically binds to Annexin V. Total microparticles were gated first by size (<1 µm) using size reference beads and thereafter gated for Annexin V positive events (Annexin V+).

Of the 42 patients originally recruited into the study, only 39 were fully studied with samples obtained pre-treatment and post-treatment. In *Figure 2*, we provide results for all initially recruited 42 patients from the pre-treatment group, however, studies with post-treatment data reflect 39 patients.

A similar number of Annexin V+ microparticles was found in plasma from cancer patients before and after treatment with VEGFi indicating that the total number of circulating microparticles was unchanged by VEGFi treatment (*Figures 1B*, *C* and *2A*). However, there were differences in the sub-sets of microparticles following treatment. As shown in *Figures 1D* and *2B*, the number of ECMPs as determined by CD31+CD41- labelling was increased following treatment, whereas the number of PMPs, determined as CD31+CD42+ labelling (Supplementary material online, *Figure S1*) was unchanged. We also evaluated expression of VEGFR2 in ECMPs and PMPs. VEGFi treatment did not influence expression of VEGFR2 in ECMP or PMP (Supplementary material online, *Figure S1A* and *C*). Phosphatidylserine expression in Annexin V+ MPs and the mean size of MPs was not different between groups (Supplementary material online, *Figure S2A* and *B*). The number of data points shown in Supplementary material online, *Figure S1* is less than that in other figures because not all samples were positive for CD31, CD42b, or VEGFR2, which means that not all MPs isolated from our patient’s blood samples expressed VEGFR2, or they may have been derived from platelets or ECs.

### 3.2 Interplay between microparticles and the ET-1 system

**Figure 3:**
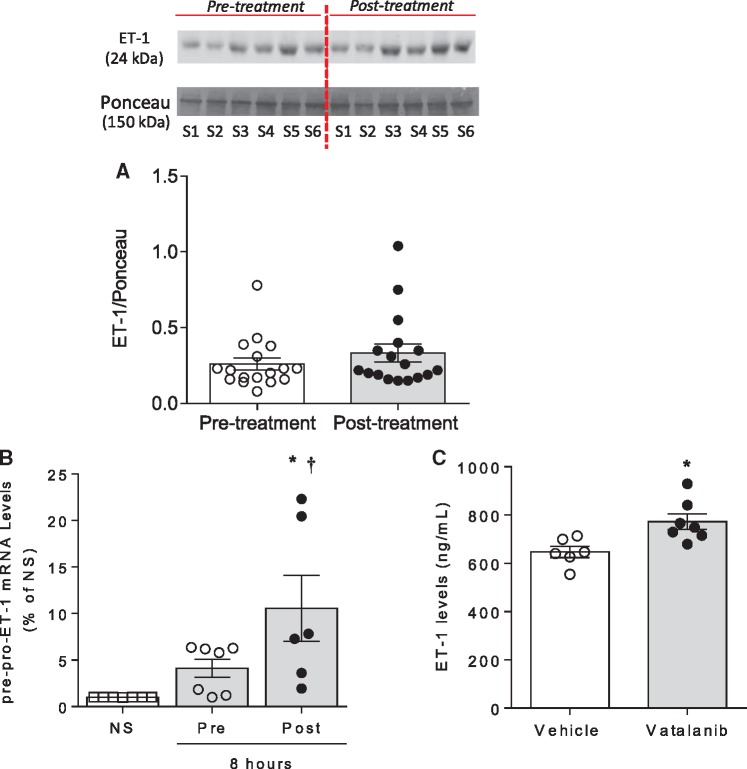
Post-treatment MPs increase pre-pro-ET-1 gene expression in HAEC. (*A*) Graph represents the expression of ET-1 assessed by immunoblotting in plasma isolated MPs from patients pre-treatment and post-treatment with VEGFi (*n* = 17). The image shows six samples from one patient and is representative of the 17 patients, pre-treatment and post-treatment. (*B*) Pre-pro-ET-1 mRNA levels assessed by qPCR in HAEC exposed to total pre-treatment (grey bar) and post-treatment (black bar) MPs for 8 h and non-stimulated (NS, white bar) HAEC (*n* = 6–7). (*C*) ET-1 levels measured by ELISA in media from HAEC stimulated with vehicle or vatalanib (VEGFi) for 8 h (*n* = 6–7). Results represent the mean ± SEM. Data were analysed using *t*-test or one-way ANOVA followed by a *post hoc* Tukey test when appropriate. *P < 0.01 vs. NS, ^†^*P* < 0.05 vs. pre-treatment. S, subject.

ET-1 is a potent vasoconstrictor and increased levels of ET-1 have been observed in patients and experimental animal models treated with VEGFi.^26–29^ In this context, ET-1 has been implicated in vascular dysfunction and hypertension in cancer patients treated with VEGFi. To explore whether microparticles act as carriers for ET-1, we assessed ET-1 expression in circulating microparticles from cancer patients before and after VEGFi treatment. As shown in *Figure 3A*, ET-1 is present in microparticles, but levels were unaffected by VEGFi. We next explored the possibility that microparticles influence the ET-1 system in effector endothelial cells. As shown in *Figure 3B*, HAEC stimulated with isolated microparticles post-treatment increased pre-pro-ET-1 gene expression, a response which was not observed in non-stimulated cells or HAEC stimulated with pre-VEGFi treatment microparticles (*Figure 3B*). To assess direct effects of VEGFi on endothelial cells, we treated HAECs with vatalanib. As shown in *Figure 3C*, vatalanib significantly increased production of ET-1.

### 3.3 Microparticles from VEGFi-treated cancer patients influence endothelial cell production of ROS and NO through ET-1-dependent processes

**Figure 4:**
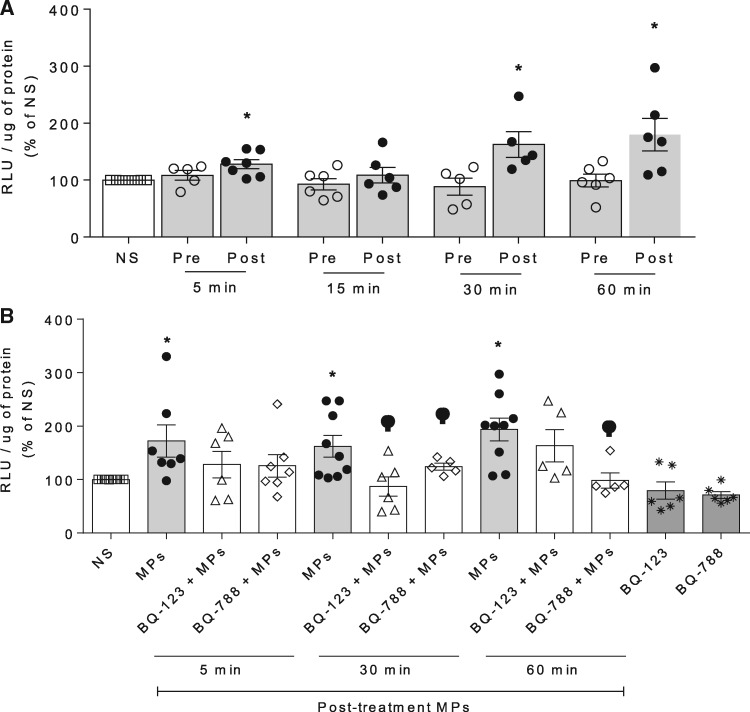
MPs increase ROS production in HAEC, which is attenuated by ET-1 receptors antagonism. NADPH-stimulated $O_{2}^{\text{−}}$ production was assessed by lucigenin assay in (*A*) HAEC exposed to total pre-treatment and post-treatment MPs for 5, 15, 30, and 60 min (*n* = 5–6) and (*B*) HAEC exposed to post-treatment MPs for 5, 30, and 60 min in presence or absence of BQ123 (ETA antagonist, 1 µmol/L) or BQ788 (ET_B_ antagonist, 1 µmol/L) (*n* = 5–9). Results represent the mean ± SEM. Data were analysed using one-way ANOVA followed by a *post hoc* Tukey test. *P < 0.05, **P < 0.01, ***P < 0.001 vs. NS; ^φ^*P* < 0.05 vs. post-treatment.

Human endothelial cells exposed to post-VEGFi treatment microparticles showed an increase in NADPH-dependent O_2_^−^ generation (*Figure 4A*). These effects were inhibited by BQ123 and BQ788, antagonists of ETA and ETB receptors, respectively (*Figure 4B*). No ROS effects were observed in HAEC stimulated with pre-VEGFi treatment microparticles.

**Figure 5:**
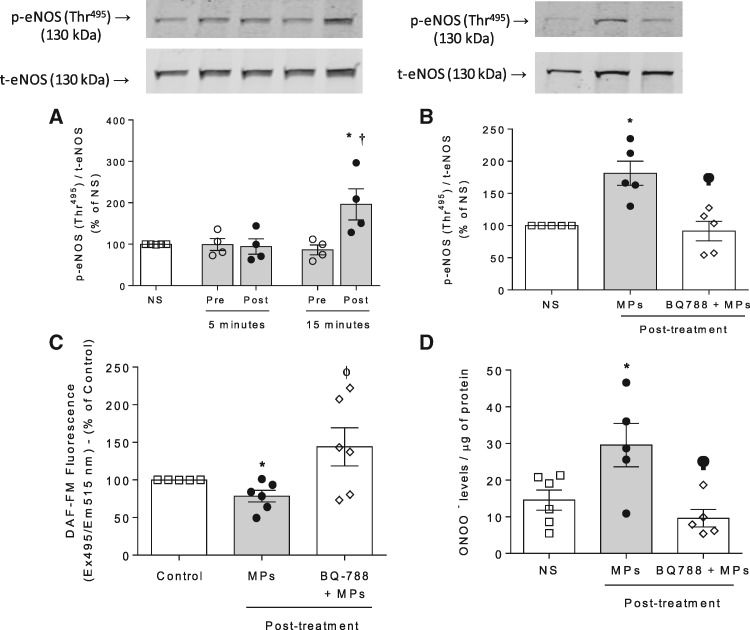
MPs reduce eNOS/NO signalling and increases ONOO^−^ in HAEC via ET_B_ receptor. (*A*) Phosphorylation of eNOS (Thr^495^) was determined by immunoblotting in non-stimulated HAEC or cells exposed to total MPs (10^6^ MPs/mL) isolated from patients’ plasma pre-treatment and post-treatment with VEGFi for 5 and 15 min (*n* = 4). (*B*) eNOS phosphorylation was assessed in HAEC stimulated with total post-treatment MPs (15 min). Cells were also pre-treated with BQ788 (ET_B_ antagonist, 1 µmol/L), added 30 min before the stimulation with MPs. Values were normalized by total eNOS expression (*n* = 5). (*C*) Nitric oxide (NO) production was determined by DAF2-DA fluorescence in HAEC exposed to total post-treatment MPs in presence and absence of BQ788 (*n* = 6). (*D*) Peroxynitrite (ONOO^−^) levels were determined by nitrotyrosine ELISA in HAEC exposed to total post-treatment MPs in presence and absence of BQ788 (*n* = 5–6). Values from DAF2-DA and nitrotyrosine ELISA were normalized by protein amount. Results represent the mean ± SEM. Data were analysed using one-way ANOVA followed by a *post hoc* Tukey test. *P < 0.05 vs. NS; ^†^*P* < 0.05 vs. pre-treatment; ϕ vs. MPs.

In HAECs stimulated with post-VEGFi treatment microparticles phosphorylation of the inhibitory motif (Thr^495^) of eNOS was increased 15 min after stimulation (*Figure 5A*). This was associated with a reduction in NO production in HAEC (*Figure 5C*). Pre-exposure of HAEC to BQ788 (*Figure 5B* and *C*) inhibited microparticle-induced effects on eNOS phosphorylation and NO production. In addition, post-VEGFi treatment microparticles promoted an increase in ONOO^−^ levels in HAEC, which was reduced by the ETB receptor antagonist (*Figure 5D*).

### 3.4 Microparticles from VEGFi-treated cancer patients modulate pro-inflammatory signalling in endothelial cells

**Figure 6:**
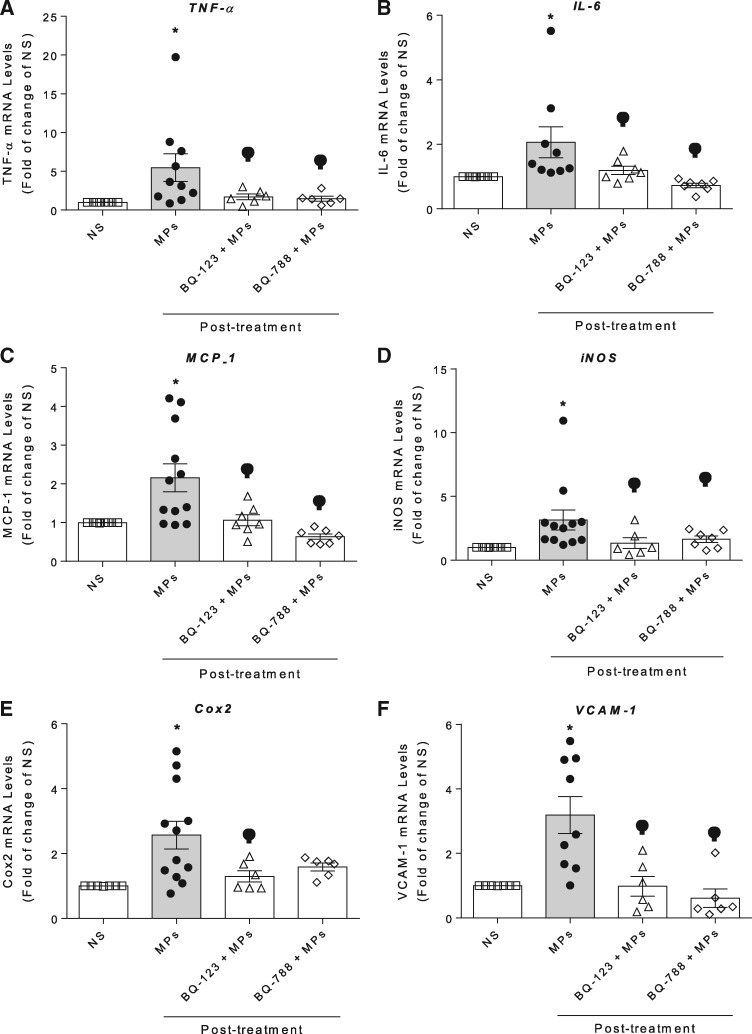
MPs increases inflammatory markers expression via ETB and ETA receptors in HAEC. mRNA expression of TNF-α, IL-6, MCP-1, iNOS, COX-2, and VCAM-1 was determined by real-time PCR in HAEC exposed to total post-treatment MPs in presence or absence of BQ123 and BQ788 pre-incubated 30 min before the stimulation with MPs. PCR values were normalized by GAPDH mRNA expression (*n* = 6–12). Results are represented as mean ± SEM. Data were analysed using one-way ANOVA followed by a *post hoc* Tukey test. *P < 0.05 vs. NS; ^φ^*P* < 0.05 vs. post-treatment.

Human endothelial cells stimulated with microparticles obtained from cancer patients post-VEGFi treatment induced a significant increase in gene expression of pro-inflammatory markers including TNF-α, IL-6, MCP-1, iNOS, COX-2, and VCAM-1 (*Figures 6A–F*). These effects were reduced in HAEC pre-exposed to BQ123 and BQ788 (*Figure 6*), indicating that the pro-inflammatory responses induced by post-VEGFi treatment microparticles are mediated through ET-1-dependent processes.

## 4. Discussion

Major findings from the present study demonstrate that, in cancer patients treated with VEGFi, (i) circulating levels of endothelial microparticles, but not PMP, are increased, (ii) microparticles express VEGFR2 and carry ET-1, (iii) microparticles undergo a functional change to a pro-inflammatory phenotype, and (iv) microparticles stimulate endothelial cell signalling and ET-1 production through ET-1-dependent processes. Our study suggests that in cancer patients, antiangiogenic drugs, such as VEGFi, promote endothelial dysfunction, and formation of endothelial microparticles, which are biologically functional units that induce endothelial cell activation and inflammation. This feedforward system seems to be mediated through increased production of endothelial-derived ET-1. Our novel findings define a new mechanism involving interplay between microparticles, the ET-1 system and endothelial cell pro-inflammatory and redox signalling, which may be important in cardiovascular toxicity and hypertension associated with VEGFi anti-cancer treatment.

Microparticles are increasingly being recognized as important bioactive effectors in many pathologic processes, such as cardiovascular^15^ and inflammatory^30^ diseases, preeclampsia,^31^ and cancer.^32^ Growing evidence shows that ECMPs play a crucial role in cardiovascular diseases by causing endothelial injury through processes involving oxidative stress and impaired vasorelaxation.^33^^,^^34^ Here we found that, in cancer patients treated with VEGFi, circulating levels of ECMPs were increased, suggesting that this may reflect underlying endothelial damage caused by VEGFi treatment. This is supported by previous studies, which demonstrated that anti-angiogenic VEGFi are associated with endothelial dysfunction and vascular injury.^26^^,^^35–37^ We previously showed that vatalanib (VEGFi) causes vascular dysfunction through redox-sensitive processes, which is translated to endothelial dysfunction, vascular hypercontractility, and cardiovascular and renal oxidative stress.^10^ The fact that the total number of microparticles as well as the PMP fraction were not altered by VEGFi indicates that effects are cell-specific and that not all cell types undergo microparticle shedding in response to treatment.

Microparticles also act as transport vehicles for a wide range of proteins, ligands, growth factors, receptors,^13^^,^^34^ and micro RNA (miRs).^38^ Our study demonstrated the novel findings that microparticles also carry ET-1, but more importantly, we have shown that VEGFi treatment induces a phenotypic change of microparticles so that they induce endothelial cell signalling through ET-1-dependent pathways. In support of these findings, previous studies demonstrated that tyrosine kinase inhibitor-induced hypertension is associated with increased circulating levels ET-1 levels.^26–29^ However, the source of ET-1 in those studies was unclear.

Mechanisms underlying the interaction between microparticles and effector target cells are complex and might be related to diffusion through the plasma membrane or via receptor or integrin interaction,^39^^,^^40^ followed by internalization of the microparticles into target cell. Additionally, microparticles can release their cargo into the extracellular space^13^^,^^41^^,^^42^ and mediate effects through a paracrine-like manner. For example, neutrophil-derived microvesicles carry cytokines, which function as pro-inflammatory mediators.^43^ Cancer cell-derived microparticles release metalloproteases and promote tumour invasion and metastases^44^^,^^45^ and PMPs transfer miR-142 into endothelial cells causing endothelial dysfunction.^38^ Regarding our findings, ET-1 containing microparticles may influence endothelial cell function by delivering ET-1 to effector cells, where it mediates actions through endothelial cell ETAR and ETBR as well as by stimulating *de novo* endothelial production of ET-1.

Endothelial dysfunction is associated with oxidative stress^46^^,^^47^ and our group has already demonstrated that microparticles themselves stimulate endothelial ROS formation and inflammatory responses, processes that are up-regulated in hypertension.^16^^,^^48^ In the current study, only microparticles from VEGFi-treated patients caused an increase in endothelial ROS production that promotes endothelial dysfunction and inflammation. These findings suggest that VEGFi treatment causes endothelial dysfunction and shedding of microparticles and that the phenotype of these cell-derived elements exhibit a pro-inflammatory phenotype. Other studies also demonstrated that microparticles from cells exposed to stressed conditions such as hyperglycaemia and angiotensin II stimulation, induce an increase in O_2_˙ production in endothelial cells^16^ and monocytes.^49^ These phenomena have also been linked to eNOS uncoupling,^26^ which further increases ROS generation. Moreover, microparticles from VEGFi-treated patients reduced endothelial eNOS activation and NO levels, processes linked to ETBR. ET-1/ETBR signalling typically causes endothelial vasodilation via NO and prostacyclin production.^50^ However, in the context of VEGFi, where microparticles promote an oxidative milieu, the protective effects of ETBR may shift to an injurious scenario with increased production of ONOO^−^, which is highly unstable and damaging. In support of this notion, in patients with obstructive sleep apnoea syndrome microparticles induce angiogenesis through a mechanism involving endothelial-derived ET-1/ETBR.^51^

Our study clearly demonstrates that VEGFi induces a change in microparticles to a pro-inflammatory phenotype as evidenced by the significant increase in expression of endothelial pro-inflammatory mediators including IL-6, TNF-α, MCP-1, Cox2, VCAM-1, and iNOS. Other studies have also shown that microparticles from various cell types including monocytes, platelets and endothelial cells, promote inflammation and apoptosis.^52–58^ Some of these processes are mediated by ROS and, as we show, the ET-1 system may also be important. Accordingly, it is possible that drugs targeting the ET-1 system may protect against the injurious vascular effects of VEGFi.

While we have clearly demonstrated that VEGFi treatment influences the formation and phenotype of microparticles in cancer patients, our study does not provide any insights regarding underlying cardiovascular risk factors, including hypertension, which may impact microparticle status. We are also unable to comment on the effect of VEGFi treatment on the ‘proteome’ of microparticles, but such information would provide insights on molecular mechanisms and signalling associated with the phenotypic changes after treatment. In addition, our study was not designed to assess relationships between microparticles and blood pressure in VEGFi-treated patients, but this is certainly an interesting notion that warrants further study.

**Figure 7:**
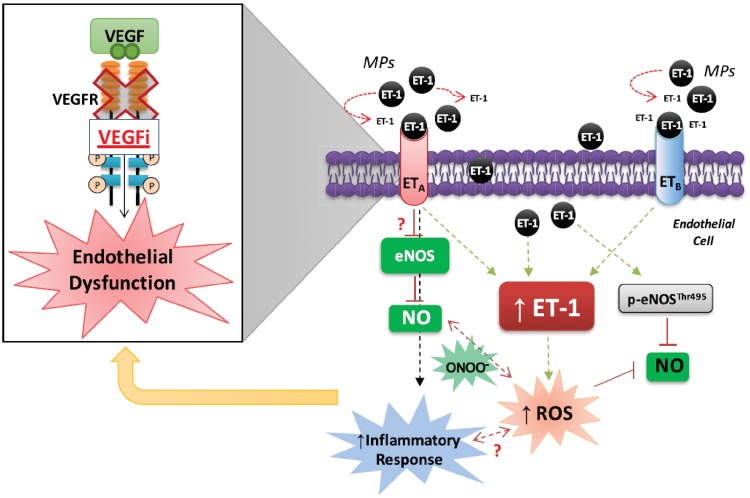
Putative mechanisms involving ET-1 system whereby MPs released under VEGFi treatment causes endothelial cell dysfunction. VEGF/VEGFR inhibition causes an increase in ECMPs release which suggests an endothelial injury. In addition to carry ET-1, MPs induce endothelial cells damage by increasing ET-1 production, inflammation and ROS production through ETA and ETB activation but also by down-regulating eNOS/NO signalling. The dysregulation of endothelial cell function induced by MPs may induce vascular tone alterations and endothelial dysfunction, and may explain, at least partially, molecular mechanisms underlying VEGFi-associated hypertension.

In summary, we show that VEGFi treatment in cancer patients promotes formation of pro-inflammatory MPs that also act as vehicles to transport ET-1 to effector target cells, where it promotes ET-1 production, ROS production and inflammation. These processes, which are mediated through ET receptors, suggest a feedforward system where MP-containing ET-1 stimulate the endothelial ET-1 system, which in turn influences ROS and NO generation (*Figure 7*). Our novel findings define a novel molecular mechanism involving interplay between microparticles, the ET-1 system and endothelial cell pro-inflammatory and redox signalling, which may be important in cardiovascular toxicity and hypertension in cancer patients treated with VEGFi.

## 5. Perspectives and significance

VEGFi are very effective anti-cancer drugs, however, they cause unwanted cardiac and vascular toxicities through ill-defined mechanisms. Our new findings demonstrate that microparticles are potential biomarkers of VEGFi-induced endothelial injury and may be important mediators of ET-1-mediated redox- and pro-inflammatory endothelial cell signalling. These findings identify a putative mechanism whereby VEGFi-induced endothelial damage begets further endothelial damage, processes that may contribute to cardiovascular complications in VEGFi-treated cancer patients.

## Supplementary Material

Supplementary Data
