# Supplementary material for: Microparticles from vascular endothelial growth factor pathway inhibitor-treated cancer patients mediate endothelial cell injury
Source: Cardiovasc Res. 2019 Feb 11;115(5):978–88. doi: 10.1093/cvr/cvz021 (PMC6452312; doi:10.1093/cvr/cvz021)
Supplement: Supplementary Data [file cvz021_supplementaty_data.docx]

**SUPPLEMENTATY DATA**

**Short title:** MPs mediates endothelial cell injury in cancer patients

Karla B Neves^1^, Francisco J Rios^1^, Robert Jones ^2, 3, 4^, T R Jeffry Evans ^2,4^, Augusto C Montezano^1^, Rhian M Touyz^1^.

^1^Institute of Cardiovascular and Medical Sciences, University of Glasgow, Glasgow - UK

^2^Beatson West of Scotland Cancer Centre, Glasgow - UK

^3^Cancer Research UK Glasgow Clinical Trials Unit, Glasgow - UK

^4^Institute of Cancer Sciences, University of Glasgow, Glasgow - UK

**Corresponding author:**

Rhian M Touyz, MD, PhD

Institute of Cardiovascular and Medical Sciences

University of Glasgow

126 University Place

Glasgow G12 8TA

Email address: rhian.touyz@glasgow.ac.uk

Telephone number: 014 1330-7775

**SUPPLEMENTARY FIGURES**

**Supplemental Figure S1. ECMPs and PMPs express VEGFR2.** (A) Scatter plot graph representing VEGFR2-expressing ECMPs in plasma pre- and post-treatment with VEGFi (n=34 to 39). (B) Representative of PMPs events identified pre- and post-treatment with VEGFi (n=23 to 27). (C) Scatter plot graph representing VEGFR2-expressing PMPs pre- and post-treatment with VEGFi (n=22 to 26). Results represent the mean ± SEM. Data were analysed using *t* test.

**Supplemental Figure S2.** (A) Phosphatidylserine expression in annexin V positive MPs was assessed by flow cytometer (n=23 to 27). (B) Mean size of MPs was measured by Nanosight^®^ in plasma samples from patients pre- and post-treatment with VEGFi (n=35). Results represent the mean ± SEM. Data were analysed using *t* test.

**SUPPLEMENTAL TABLES**

**Table S1. Clinical features of patients from whom plasma MPs were isolated pre-and post-VEGFi treatment.**

|  | **Male** | **Female** |
| --- | --- | --- |
| Sample size | 30 | 9 |
| Number of patients with renal cancer | 24 | 8 |
| Number of patients with other cancer (colorectal, oesophagus, etc) | 6 | 1 |
| Number of patients on sunitinib | 3 | 1 |
| Number of patients on pazopanib | 21 | 7 |
| Number of patients on other drugs (sorafenib, etc) | 4 | 1 |
| Number of patients on other drugs | 2 | - |
|  |  |  |

Forty two patients originally entered the study but only 39 were fully studied.

**Table S2.** Clinical parameters of patients

| **Clinical Condition** | **Number of Patients** |
| --- | --- |
| Nephrectomy | 17 |
| Transarterial chemo-embolization (TACE) | 3 |
| Prior cytotoxic chemotherapy | 1 |
| Ischemic heart disease | 6 |
| Atrial Fibrillation with no Ischemic Heart Disease | 2 |
| No history of heart diseases | 26 |
| Prior Hypertension | 17 |
| Unknown | 5 |

**Table S3.** List of human (h) primers used.

| Primer | Sense primer | | Anti-sense primer |
| --- | --- | --- | --- |
| hPre-pro-ET-1 | | GGGGATCTGAGTCTGTCCAA | CAACACACATGCTGGGAAAC |
| hTNF-α | | AGCCCATGTTGTAGCAAACC | TGAGGTACAGGCCCTCTGAT |
| hIL-6 | | AGGAGACTTGCCTGGTGAAA | CAGGGGTGGTTATTGCATCT |
| hMCP-1 | | CCCCAGTCACCTGCTGTTAT | AGATCTCCTTGGCCACAATG |
| hiNOS | | ACAAGCCTACCCCTCCAGAT | TCCCGTCAGTTGGTAGGTTC |
| hCOX2 | | TGAGCATCTACGGTTTGCTG | TGCTTGTCTGGAACAACTGC |
| hVCAM-1 | | GCACCTTTCTGGAAATGCAA | GTCTCCAATCTGAGCAGCAA |
| hGAPDH | | GAGTCAACGGATTTGGTCGT | TTGATTTTGGAGGGATCTCG |
